# Stability of olfactory behavior syndromes in the *Drosophila* larva

**DOI:** 10.1038/s41598-023-29523-x

**Published:** 2023-02-10

**Authors:** Seth R. Odell, Nicholas Zito, David Clark, Dennis Mathew

**Affiliations:** 1 Integrative Neuroscience Program, University of Nevada, Reno, NV 89557 USA; 2 Department of Biology, University of Nevada, Reno, NV 89557 USA

**Keywords:** Sensory processing, Olfactory system

## Abstract

Individuals of many animal populations exhibit idiosyncratic behaviors. One measure of idiosyncratic behavior is a behavior syndrome, defined as the stability of one or more behavior traits in an individual across different situations. While behavior syndromes have been described in various animal systems, their properties and the circuit mechanisms that generate them are poorly understood. We thus have an incomplete understanding of how circuit properties influence animal behavior. Here, we characterize olfactory behavior syndromes in the *Drosophila* larva. We show that larvae exhibit idiosyncrasies in their olfactory behavior over short time scales. They are influenced by the larva’s satiety state and odor environment. Additionally, we identified a group of antennal lobe local neurons that influence the larva’s idiosyncratic behavior. These findings reveal previously unsuspected influences on idiosyncratic behavior. They further affirm the idea that idiosyncrasies are not simply statistical phenomena but manifestations of neural mechanisms. In light of these findings, we discuss more broadly the importance of idiosyncrasies to animal survival and how they might be studied.

## Introduction

Animal behavior is notoriously variable. Individual variations and idiosyncratic behaviors are found in vertebrate and invertebrate systems^1–3^. Even in humans, personality differences among genetically identical twins determine their individual responses to stimuli^4,5^. A measure of individuality or idiosyncratic behavior is a behavior syndrome, defined as the stability of one or more behavior traits in an individual under different situations^1^. For instance, aggressive behavior (trait 1) of an individual stickleback fish towards conspecifics (situation 1) is often correlated with its feeding behavior (trait 2) under risk of predation (situation 2)^6^. Another example of a behavior syndrome is the correlation between voracity (trait 1) in a juvenile fishing spider (situation 1) and its voracity as an adult (situation 2)^7^. Simple variations in behavior among individuals of a genetically isogenic population challenge a common assumption in behavior studies that all individuals are behaviorally equivalent. However, the stability of behavior syndromes further suggests that there is covariation among behavior traits^8,9^. Scientists now appreciate that individuality and idiosyncratic behavior are not simply statistical phenomena but manifestations of neural circuit properties. Indeed, idiosyncratic behaviors may have significance for species survival^10,11^.

However, attempts to understand the origins of idiosyncratic behavior have traditionally been based on evolutionary and developmental frameworks^11^. For instance, research on the stability of behavior syndromes has focused chiefly on developmental situations, e.g., behavior in the larval stage vs. adults^7,12–14^. Such approaches have provided and will continue to provide novel insights. But lately, researchers have discovered that noisy, transient neural mechanisms within short developmental timescales are also important for generating idiosyncratic behaviors^15,16^. Some of these studies suggest that cross-situational stability within shorter developmental timescales is weaker than stability across longer timescales^17,18^. What is not understood is whether the stability of idiosyncratic behavior is influenced by an animal’s internal state, external environment, or different circuit mechanisms. If we better understood the fundamental properties of idiosyncratic behavior among individuals and the origins of idiosyncrasies in the olfactory circuit, we would then be able to clarify the fundamental ways in which circuit properties influence olfactory behavior.

To understand the fundamental properties of idiosyncratic behavior among individual animals, we need to extensively analyze behavior idiosyncrasies, preferably using a well-studied behavior paradigm, in a genetically tractable model organism with a simple neural circuit. Studying olfactory behavior in the common household fruit fly, *Drosophila* presents opportunities to do so. In *Drosophila*, untrained odor preferences vary widely among individuals but rarely fluctuate when the same flies are repeatedly tested. Even though individual odor preferences may be modified by Pavlovian conditioning, the rank order of preference was preserved across individuals even after training^19^. In this study, although the authors observed individuality in fly olfactory behavior, they did not test its stability or the factors influencing them. Other studies suggested that variability in morphological and physiological properties of olfactory neurons might provide a basis for idiosyncratic olfactory behavior among individual flies. Indeed, there is considerable imprecision in connections between first- and second-order olfactory neurons as well as variability in physiological properties among a genetically defined class of local neurons in the antennal lobe^20,21^. Furthermore, a recent study suggested that the degree of fly-to-fly variability in olfactory preference is affected by neuromodulation, environmental stressors such as nutrition, and activity of local neurons in the antennal lobe, an olfactory processing center^22^. Thus, while idiosyncrasy in *Drosophila* olfactory behavior is well established, its properties and factors that influence them are less well characterized.

In this study, we asked whether olfactory behavior syndromes in the larvae are stable within short developmental timescales and whether this stability is influenced by specific internal and external situations. We also asked whether olfactory circuit neurons influence behavior syndromes. To address these questions, we conducted four experiments that characterized the stability of olfactory behavior syndromes in the *Drosophila* larva within a short developmental timescale. We identify specific influences on behavior syndromes and discuss the importance of studying idiosyncrasies in animal behavior. Ultimately, idiosyncratic behavior responses to odors under different situations have consequences for the animal’s survival.

## Materials and methods

### *Drosophila* stocks

A Canton-S (CS) line was used as a wild-type line in experiments 1 and 2. For optogenetic assays in experiment 3, females from a *UAS-IVS-CsChrimson *(BDSC #55135; gene expressing a red-light sensitive *ChRhodopsin*) were crossed to males from an *OrX-Gal4* (where *X* = 7a/42a/42b/45a/45b/47a/67b) (from Dr. John Carlson). Parent lines were used as control flies for these experiments. For the Gal4 screen in experiment 4, females from a * UAS-Shi*^*ts*^ (BDSC #44222) were crossed to males from seven different Gal4 lines: Acj6 (BDSC #30025), 189Y (BDSC #30817), NP3056 (BDSC #188030), 421 (BDSC #66306), 449 (BDSC #63325), Keystone (BDSC #49232), and SEZ (BDSC #48864). Flies were reared at 25 °C and 60% humidity on standard cornmeal-dextrose agar food (Genesee Scientific, #66-112).

### Odorants and other reagents

Test odorants were obtained at the highest purity available (≥ 98% purity; Sigma-Aldrich). Odors were diluted in paraffin oil (Sigma-Aldrich, #76235). Larval crawling surface for behavior experiments was prepared using high-purity Agarose (Genesee Scientific #20-102GP). Odor gradients in the behavior arena were generated by adding odor to 6 mm filter discs (GE Healthcare #2017-006) placed in the arena.

### Preparation of animals for behavior assays

Third-instar *Drosophila* larvae (~ 96 h after egg laying) were used for behavior experiments. Larvae were extracted from growth media using a high-density (15%) sucrose solution (Sigma Aldrich, #S0389). Larvae that float to the surface of the sucrose solution were separated into a 1000 mL glass beaker and washed four times with distilled water. For experiments 1 and 2, washed larvae were transferred to a 6 cm petri-dish (Falcon Scientific, #351007) containing 350 µL 0.2 M sucrose solution added to a Kim wipe. The transferred larvae were allowed to roam freely for 2 h (non-starved condition).

For optogenetic assays in experiment 3, 400 μL of all-trans-retinal (ATR) mixture was added to the larval food vial 48 h after egg-laying. ATR mixture contained 400 μM ATR dissolved in dimethyl sulfoxide (DMSO) and 89 mM sucrose dissolved in distilled water. Sucrose promotes larval feeding of ATR. ATR is a cofactor required for upregulation of *ChRhodopsin* expression^23,24^. Following ATR addition, vials were covered with Aluminum foil, and larvae were allowed to develop to the third-instar stage in the dark.

### Larval tracking

The larval tracking method was adapted from Mathew et al.^25^. Assays were performed on a 22 cm × 22 cm petri dish layered with 1.5% agarose, except when mentioned otherwise. Larvae are always placed in the middle of the plate at the start of the assay. The movement of larvae in the arena was recorded at 2.3 frames/s using a Monochrome USB 3.0 camera (Basler Ace series, JH Technologies). The camera was fitted with an IR long-pass 830 nm filter and an 8 mm F1.4 C-mount lens (JH Technologies) to image larvae under dark-field illumination with infrared LEDs (850 nm, outside the range of larval phototaxis; Environmental Lights Inc.). Each pixel in the recorded image equaled 0.119 mm^2^ of the experimental arena.

#### Analysis

Analysis of larval trajectories was adapted from Mathew et al.^25^ and Gershow et al.^26^. Larval positions in the behavior arena were extracted from video recordings. Larval trajectories were reconstructed using custom routines written in MATLAB. Trajectories were segmented into a series of ‘runs’ and ‘stops.’ Runs were defined as continuous periods of forward movement. A stop separated successive runs and was flagged when the speed dropped beneath a threshold value (unique to each larva) for more than two frames. Stops were further examined to differentiate between a ‘stop’ and a ‘head sweep.’ A head sweep was flagged if a larva was stopped and its body bend angle was greater than 20°^26^. ‘Run length’ and ‘run speed’ were calculated from the analysis of runs. ‘% time stopped’ was calculated from time spent during stops. ‘No. of head sweeps’ was counted for every larval trajectory. ‘Curvature’ was calculated as the total length of the larval trajectory divided by its total displacement. Finally, ‘%arena explored’ was calculated as the number of 5 mm × 5 mm squares along the trajectories divided by the total number of squares available in the arena. Principal component analysis (PCA) was performed using MATLAB (RRID: SCR_001622). Variables were standardized by converting values to z-score as well as centered. A component was considered for analysis if it explained variance for more than one variable.

### Experiment 1

**Figure 1:**
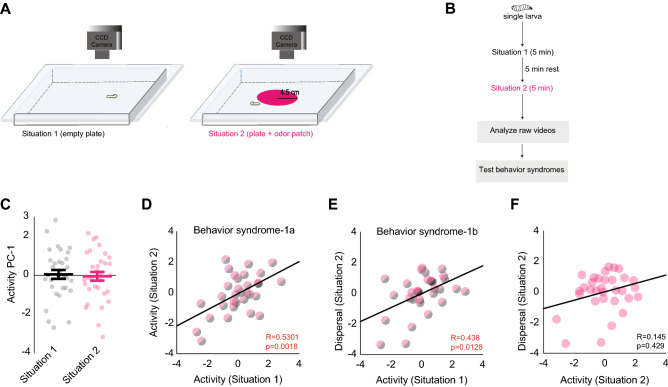
Experiment 1: Stability of olfactory behavior syndromes. (**A**) A single wild-type larva is exposed to situation 1 (left, empty plate); situation 2 (right, plate with odor patch). Larval movements are recorded by a CCD camera, n = 32. Adapted from Mathew et al.25. (**B**) A schematic of the paradigm is provided on the right. (**C**) Average activity measure for larvae in situation 1, n = 32, and situation 2, n = 32. Repeated measures t-test, p = 0.659. (**D**) Correlation between the activity of a larva in situation 1 and its activity in situation 2. p < 0.05, Pearson’s correlation, n = 31. (**E**) Correlation between the activity of larva in situation 1 and its dispersal in situation 2. p < 0.05, Spearman’s correlation, n = 32. (**F**) Correlation between the activity of a larva in situation 2 and its dispersal in situation 2. p = 0.429, Spearman’s correlation, n = 31.

In experiment 1, a single wild-type larva was subjected to two five-minute tracking trials: the first trial was performed in an empty agarose arena, and the second trial, with a 5-min interval in between, was conducted in an odor arena. The odor arena is an agarose arena, in which a 4.5 cm radius patch was removed from the center and replaced with a similar patch of agarose mixed with 10^–2^ ethyl acetate (vol: vol) (Fig. 1A). We chose this odor because *Drosophila melanogaster* larvae show strong attractive behavior to ethyl acetate^27^. Between each trial, the larva was allowed to rest for five minutes (Fig. 1B).

#### Analysis

Movements of individual larvae were tracked in both situations using a CCD camera, and behavior metrics were extracted from the resulting videos. We analyzed 32 larval tracks in both situations. ‘Max distance from the center of odor patch’ was calculated as the maximum distance between the center of the plate and larva. ‘Time to leave odor patch’ was calculated as the time required for a larva to reach 4.5 cm from the center of the plate. If a larva never reached this distance, this value was set to 300 s. Larval activity (behavior trait) was measured as a composite of three behavior metrics: ‘mean run speed,’ ‘% plate explored,’ and ‘% time stopped.’ A principal component analysis (PCA) was carried out based on the three behavior metrics. The principal axes collapsed into a single component, labeled ‘activity’ (Supplementary Fig. 1A). A high value on this component indicated high activity, and a low value indicated low activity. Based on the variable loads, high ‘activity’ indicated higher ‘run speed,’ ‘more % plate explored,’ and smaller ‘% time stopped.’ We used a similar approach to measure larval dispersal in the odor arena. We evaluated two behavior metrics: ‘maximum distance traveled from the center of odor patch’ and the ‘time to leave odor patch.’ PCA was performed. The principal axes collapsed into a single component labeled ‘dispersal’ (Supplementary Fig. 1B). High ‘dispersal’ indicated higher values for ‘maximum distance traveled from the center of odor patch’ and smaller values for the ‘time to leave odor patch.’ Next, we considered two olfactory behavior syndromes: In behavior syndrome-1a, the stability of one behavior trait (activity) of individual third-instar larvae was compared across two different situations: situation 1 was the empty agarose arena; situation 2 was the odor arena. In behavior syndrome-1b, the stabilities of two behavior traits (activity and dispersal) were compared across situation 1 (empty arena) and situation 2 (odor arena).

### Experiment 2

**Figure 2:**
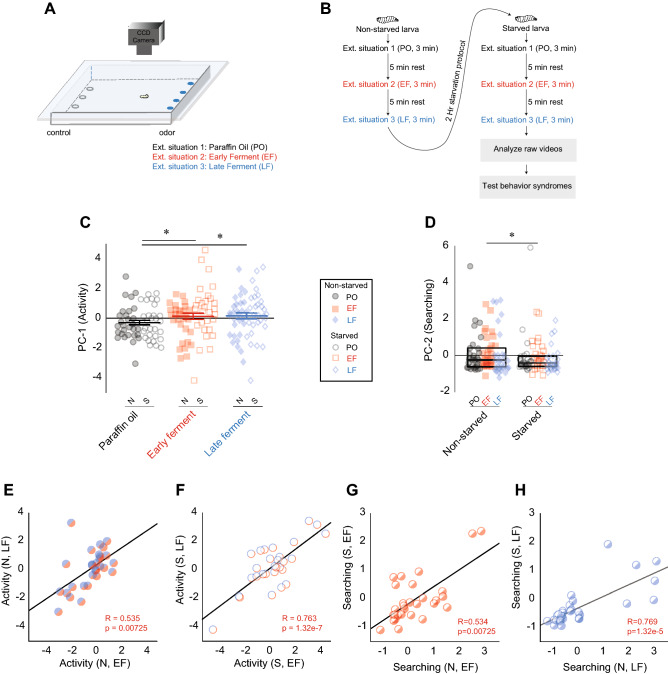
Experiment 2: Effect of internal and external situations. (**A**) A single non-starved larva is sequentially exposed to three situations: situation 1 (PO: no odor); situation 2 (EF: early ferment odor); situation 3 (LF: late ferment odor). The larva is then starved for 2 h and again exposed to the same situations. Larval movements are recorded by a CCD camera, n = 30. Adapted from Mathew et al.^25^. (**B**) A schematic of the paradigm is provided on the right. (**C**) Average activity measure for non-starved (filled shapes) and starved (empty shapes) larvae in situation 1 (black circles), situation 2 (red squares), and situation 3 (blue diamonds). *p = 0.048 (PO vs EF) and *p = 0.0328 (PO vs LF), ANOVA. (**D**) Average searching measure for the same set of larvae. *p = 0.005, non-parametric ANOVA. (**E–H**) Correlation examples: (**E)** between the activity of non-starved larvae in situation 2 and its activity in situation 3. p < 0.025, Pearson’s correlation, n = 28; (**F**) between the activity of starved larvae in situation 2 and its activity in situation 3. p < 0.0001, Pearson’s correlation, n = 28; (**G**) between searching of larvae in situation 2 when non-starved and its searching in the same situation when starved. p < 0.025, Spearman’s correlation, n = 28; (**H**) between ‘searching’ of larvae in situation 3 when non-starved and its ‘searching’ in the same situation when starved. p < 0.025, Spearman’s correlation, n = 28.

In experiment 2, we examined the stability of larval olfactory behavior syndromes under different internal (starved, non-starved) and external situations (no odor, early-ferment odor, late-ferment odor). Before performing the assay, larvae were subjected to either starved (S—provided dH_2_O for 2 h) or non-starved (N—provided 0.2 M sucrose for 2 h) conditions. A single larva (S or N) was sequentially subjected to three external situations in a 22 cm × 22 cm agarose arena for 3 min each: External situation 1 was the absence of odor (PO); External situation 2 was the presence of an odor blend similar to early yeast (*S. cerevisiae*) ferment of grapes (EF—acetal: acetic acid at a ratio of 1:1); External situation 3 was the presence of an odor blend similar to the late yeast ferment of grapes (LF—acetal: acetic acid at a ratio of 5:1) (Fig. 2A)^28^. We chose these odor situations because both adults and larvae of *D. melanogaster* are attracted to volatiles generated from the fermentation of grapes by *S. cerevisiae*^25,29^. Odor gradients were generated in the arena by adding test odor to five filter discs placed equidistant from each other along one side of the arena. The diluent (paraffin oil) was added to five filter discs along the opposite side. Each time a larva was transferred between situations, a fresh agarose arena was used. Between each trial, the larva was removed from the arena and allowed to rest for 5 min. Following the third trial, the larva was transferred to a 6 cm petri dish containing 350 μL of dH_2_O, added to a Kim wipe, and allowed to roam freely for 2 h (starved condition). It was then exposed to the above three trials again (Fig. 2B). Direction of the odor gradient in the arena was altered for each trial.

#### Analysis

In each situation, larval movements were tracked. We analyzed 29 larval tracks in each of the six situations. Five behavior metrics were extracted: ‘run speed,’ ‘run length,’ ‘total curvature,’ ‘no. of head sweeps,’ and ‘% time stopped.’ PCA was performed. The principal axes of these five metrics collapsed into two components, PC-1 and PC-2 (Supplementary Fig. 2A–C). Since ‘run speed’ and ‘run length’ values loaded positively and ‘% time stopped’ values loaded negatively onto PC-1, we refer to this component as ‘activity.’ High values of PC-1 (Activity) indicate a larva with a higher run speed and run length and spent less time at a stop. Since ‘no. of head sweep’ and ‘total curvature’ values loaded positively onto PC-2, we refer to this component as ‘searching.’ Head sweeps, and more meandering runs (high values for curvature—overall run-length/actual displacement) are typically associated with searching behaviors^30^. High values of PC-2 (Search) indicate a larva that had a higher total number of head sweeps and a more meandering track) high values for curvature—overall length/displacement). For the behavior syndrome in experiment 2, we considered the stabilities of ‘activity’ and ‘searching’ traits in individual larvae across six situations: S-PO, S-EF, S-LF, N-PO, N-EF, and N-LF.

### Experiment 3

**Figure 3:**
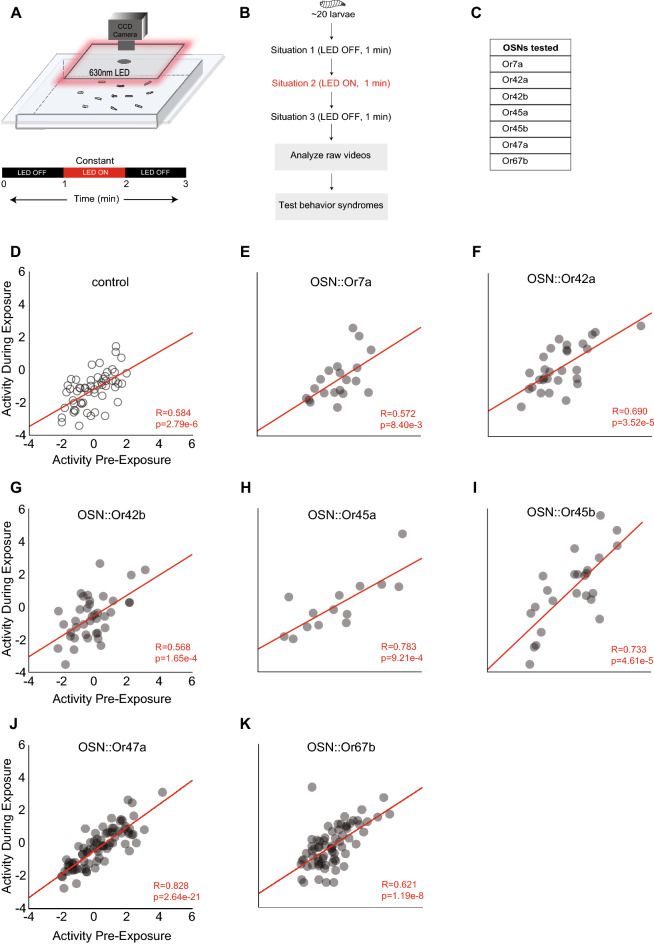
Experiment 3: Influence of sensory neurons on olfactory behavior syndromes. (**A**) ~ 20 transgenic larvae, each expressing *ChRhodopsin* in a single pair of olfactory sensory neurons (OSNs), are allowed to roam freely on an agarose arena. OSNs expressing *ChRhodopsin* are activated by shining red light (630 nm) on the arena. Larvae are subjected to three situations: situation 1 (pre-exposure: lights OFF, 1 min.); situation 2 (during-exposure: lights ON, 1 min.); situation 3 (post-exposure: lights OFF, 1 min.). Larval movements are recorded by a CCD camera. Adapted from Clark et al.^31^. The assay is repeated 5 times for each set of OSNs targeted and control animals. (**B**) A schematic of the paradigm and (**C**) a list of seven OSNs targeted in this study are provided on the right. (**D-K**) Correlations between the activity of larva in situation 1 (pre-exposure) and its activity in situation 2 (during exposure) are shown for (**D**) control larvae, p = 2.79e^-6^, n = 55, and for larvae in which the following OSNs are activated: (**E**) OSN::Or7a, p = 8.40e^-3^, n = 20; (**F**) OSN::Or42a, p = 3.25e^-5^, n = 28; (**G**) OSN::Or42b, p = 1.65e^-4^, n = 39; (**H**) OSN::Or45a, p = 9.21e^-4^, n = 14; (**I**) OSN::45b, p = 4.61e^-5^, n = 25; (**J**) OSN::Or47a, p = 2.64e^-21^, n = 15; (**K**) OSN::Or67b, p = 1.19e^-8^, n = 16. Pearson’s correlation.

In experiment 3, we considered the impact of activating individual first-order olfactory sensory neurons (OSNs) on the stability of a larval olfactory behavior syndrome. An optogenetic technique, previously developed in our lab, was used to spatially and temporally activate single larval OSNs and simultaneously record larval movement^31,32^. Briefly, the UAS-GAL4 system was used to express red light-responsive channel rhodopsin (*CsChrimson*) separately in seven different OSNs. As 20 transgenic larvae crawled on an empty agarose arena, *CsChrimson* expressing OSNs were activated by shining red light (630 nm wavelength, 1.3 W/m^2^ intensity) on the arena for 1 min. Each trial had three conditions and lasted three minutes: the first minute (pre-exposure) had red-light stimulus OFF; the second minute (during exposure) had red-light (constant) stimulus ON; the third minute (post-exposure) had red-light stimulus OFF (Fig. 3A, B). We chose to test a representative sample of seven of the 21 larval OSNs (Fig. 3C). The OSNs tested included OSN::7a, OSN::42a, OSN::42b, OSN::45a, OSN::45b, OSN::47a, and OSN::67b. The selected OSNs included OSNs activated by attractive odors (e.g., OSN::42a, OSN::42b, OSN::47a)^25^ as well as OSNs activated by aversive compounds (e.g., OSN::7a)^33^. No odors were present during these assays.

*Analysis*: Data analysis for this experiment was conducted as described before^31^. Movements of ~ 20 larvae were simultaneously tracked pre-, during, and post-light exposure (Fig. 3B). We analyzed a total of 330 larval tracks (55 tracks for the control line and between 14 and 80 tracks for the experimental lines). Due to the lack of a directional odor source, only three behavior metrics were extracted: ‘mean run speed,’ ‘% arena explored,’ and ‘% time stopped.’ PCA was performed. ‘Activity’ was measured, similar to Experiment 1, as the principal component of the three metrics (Supplementary Fig. 3A–D). High values of PC-1 (Activity) indicate a larva with a higher run speed and a larva that explored more of the plate and spent less time at a stop. For the behavior syndrome in experiment 3, we compared the stability of ‘activity’ (trait) of an individual larva between three situations: situation 1 (pre-exposure: lights OFF, 1 min); situation 2 (during-exposure: lights ON, 1 min); situation 3 (post-exposure: lights OFF, 1 min).

### Experiment 4

**Figure 4:**
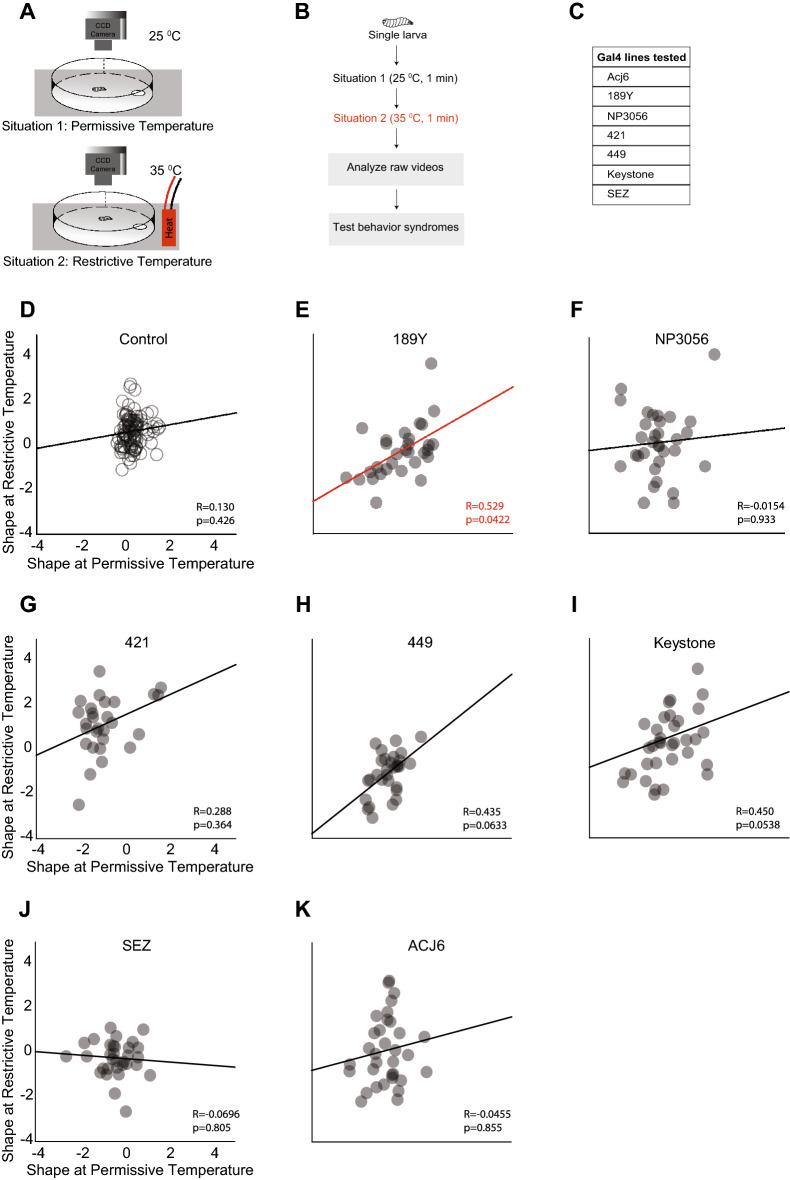
Experiment 4: Influence of antennal lobe neurons on olfactory behavior syndromes. (**A**) A single larva expressing *Shi*^*ts*^ in a set of target neurons is allowed to roam freely in a 6-cm Petri dish layered with agarose. Target neurons are inactivated by raising the temperature of the arena to 35 °C. Larval movements are recorded under situation 1 (25 °C) and situation 2 (35 °C). The assay is repeated for ~ 90 larvae in the parental control line and ~ 30 larvae for each experimental line. (**B**) A schematic of the paradigm and (**C**) a list of seven Gal4s used to target specific sets of downstream neurons are provided on the right. (**D–K**) Correlations between the shape of larval movement in situation 1 (permissive temperature, 25 °C) and restrictive temperature, 35 °C) are shown (**D**) control larvae, p = 0.426, n = 93 and larvae in which sets of neurons targeted by the following Gal4 lines are inactivated: (**E**) 189Y, p = 0.0422, n = 30; (**F**) NP3056, p = 0.933, n = 32; (**G**) 421, p = 0.364, n = 28; (**H**) 449, p = 0.0633, n = 30; (**I**) Keystone, p = 0.0538, n = 35; (**J**) SEZ, p = 0.805, n = 32; (**K**) Acj6, p = 0.855, n = 33.

In experiment 4, we performed a Gal4 screen to determine the role of peripheral olfactory neurons in influencing the stability of an olfactory behavior syndrome. Each of the seven tested Gal4 lines drives expression in either second-order projection neurons (Acj6) or a different set of local neurons in the antennal lobe (189Y, NP3056, 421, 449, Keystone) or in neurons of the sub-esophageal zone (SEZ) (Fig. 4C). We crossed each Gal4 line to a *UAS-Shi*^*ts*^ line. The temperature-sensitive *Shi*^*ts*^ (Shibire) gene, which encodes a temperature-sensitive Dynamin, allowed us to spatially and temporally inactivate different subsets of neurons to determine their role in influencing behavior syndrome stability^34^. An uncrossed parent line was used as a control. First, a single control or test larva was tracked on a 9-cm petri dish for one minute. Next, the larva was warmed to the restrictive temperature (35 °C), placed onto a heated dish, and tracked for an additional minute (Fig. 4A, B). We used an ethyl acetate (10^–2^ vol: vol) odor gradient in each trial.

#### Analysis

Overall, movements of ~ 300 larvae were tracked pre- and post-heat exposure (90 tracks for the uncrossed *UAS*-*Shi*^*ts*^ parent control line and between 28 and 35 tracks for each experimental line). We analyzed five behavior metrics for each track: ‘mean run speed,’ ‘mean run length,’ ‘total number of head sweeps,’ ‘total length over displacement,’ and the ‘percentage of time at a stop.’ PCA was performed. The behavior collapsed into two main components dubbed “Activity” and “Shape”(Supplementary Fig. 4A–C). A high value in the “activity” component indicates that the larva spent little time at stop and showed some combination of longer run lengths, increased head sweeps, and/or more meandering tracks. A high value in the “shape” behavior indicates a meandering track with more head sweeps with lower run speeds and lengths. For the behavior syndrome in experiment 4, we compared the stabilities of ‘activity’ and ‘shape’ traits of individual larvae before (situation-1, 25 °C) and after (situation-2, 35 °C) neuron inactivation.

#### A note on the various metrics used for analysis

The various metrics used in this study are defined based on how the different behavior parameters (run speed, curvature, etc.) covaried in the PCA analysis. The ‘activity’ metric is consistent throughout the four experiments (with minor differences) since it has similar principal component makeups. However, due to their respective principal component makeups, we needed to distinguish between the ‘search’ metric in Experiment 2 and the ‘shape’ metric in Experiment 4. A high ‘search’ value indicates a curving track with many head sweeps and is not affected by the run lengths or speed. So, a track made up of two long runs with no head sweeps would have a similar ‘search’ score as a track made up of six short runs with no head sweeps. On the other hand, ‘shape’ was influenced by the run length and speed to a large degree. So in the above example, the two tracks would have different ‘shape’ scores. Since the scoring of ‘search’ and ‘shape’ are different, they were considered as separate metrics for analysis.

### Statistics

Unless otherwise noted, statistical analyses were performed using Statistica (StatSoft; RRID: SCR_014213). An individual larva was only considered for analysis if its trajectory recorded at least two runs. Run speed and run length were averaged for each trajectory, giving one value for each individual. We performed principal component analysis (PCA) for each experiment to summarize larval behavior. The normality of principal components (PCs) was tested with the Shapiro–Wilk test. PCs describing ‘activity’ followed normal distributions. MANOVA followed by a Tukey HSD post-hoc test was used to analyze the main effect of ‘activity.’ Reported p-values indicate significance after adjustment for multiple corrections by Statistica. PC describing ‘searching’ did not follow a normal distribution. Therefore, a non-parametric analysis equivalent to a repeated-measures ANOVA was used to analyze the main effect of ‘searching’ (nparLD package in R (RRID: SCR_001905)^35^. Correlation analyses were performed in Statistica using either Pearson’s or Spearman’s correlation matrix, as appropriate. The BH method for controlling false discovery rates was used for multiple comparisons^36^.

## Results

### Stability of olfactory behavior syndromes within a short developmental timescale

Our first experiment sought to test the hypothesis that olfactory behavior syndromes remain stable over short developmental timescales. To test this hypothesis, we considered two olfactory behavior syndromes in experiment 1. First, we noted that although individual larvae showed varying levels of activity, there was no difference in the average activity of all larvae tested between the two situations (repeated measures t-test; t = − 0.446, p = 0.659) (Fig. 1C). In behavior syndrome-1a, we observed a positive correlation between activity (trait-1) of individual larvae across situation-1 (empty arena) and situation-2 (odor arena) (Pearson’s correlation; r = 0.5301, p = 0.0018) (Fig. 1D). Thus, a larva that was highly active in the absence of odor remained highly active in the presence of odor.

In behavior syndrome-1b, we noted a significant correlation between activity (trait-1) in situation 1 and dispersal (trait-2) in situation 2 (Spearman’s correlation; r = 0.438, p = 0.0128) (Fig. 1E). Thus, a larva that was highly active without odor tended to exhibit high dispersal in the presence of odor. However, this relationship was not observed when comparing the activity of larvae in situation 2 with their dispersal in situation 2 (Fig. 1F) (Spearman’s correlation; r = 0.145, p = 0.429).

In the two olfactory behavior syndromes we tested, we note that larvae show idiosyncrasies in their behavior responses across different situations. Our results suggest that olfactory behavior syndromes remain stable even within a short developmental timescale (tens of minutes during the third-instar larval stage).

### The stability of olfactory behavior syndromes depends on internal and external situations

Next, we asked whether this stability could be influenced by internal and external situations. An animal’s internal states (e.g., satiety) and external odor environments influence information processing in its neural circuits, which, in turn, determine its behavior response^37–42^. Therefore, in experiment 2, we examined a larval olfactory behavior syndrome under different internal (starved, non-starved) and external situations (no odor, early-ferment odor, late-ferment odor). First, we compared average values for activity (trait 1) and searching (trait 2) across internal and external situations. Larval ‘activity’ (trait 1) was influenced by the odor environment (ANOVA; p = 0.0235). Larval ‘activity’ in the presence of either odor blend, EF (0.132 ± 0.204) and LF (0.160 ± 0.194) was significantly higher compared to no-odor situation, PO (− 0.292 ± 0.149) (ANOVA, Tukey HSD; PO vs. EF: p = 0.048 and PO vs. LF: p = 0.0328) (Fig. 2C: compare colored shapes to grey circles). However, overall larval activity was not influenced by the animal’s satiety state (ANOVA, p = 0.199) (Fig. 2C: compare filled (N) and empty (S) shapes of the same color). On the other hand, larval ‘searching’ (trait 2) was influenced by the animal’s satiety state (non-parametric ANOVA; N vs. S: p = 0.00531) but not by the odor environment. Non-starved animals (− 0.247, − 0.616 to 0.395 [median, IQR]) showed a higher degree of searching compared to starved animals (− 0.419, − 0.603 to − 0.0510) given a particular odor environment (Fig. 2D: compare filled (N) and empty shapes (S)). Overall larval ‘searching’ was not influenced by the odor environment (Fig. 6B: compare filled and empty shapes to each other; ANOVA, p = 0.0637).

What about the stability of this behavior syndrome? We considered the stabilities of activity (trait 1) and searching (trait 2) in individual larvae across situations. Six situations were compared: S-PO, S-EF, S-LF, N-PO, N-EF, and N-LF. ‘Activity’ measure showed moderate to strong correlations between external situations (odor) (e.g., S-EF vs. S-LF: Pearson’s; r = 0.763, p = 1.47 × 10^–6^) (Supplementary Table 1A, B). Thus, independent of its satiety state, an individual with high activity in external situation 1 tended to have high activity in external situations 2 and 3. Two examples comparing N-EF with N-LF and S-EF with S-LF are shown (Fig. 2E, F). On the other hand, activity did not correlate between internal situations (satiety state) for any odor considered. For instance, the activity of a non-starved larva in situations 1, 2, or 3 did not correlate with its activity after starvation (Supplementary Table 1C). We noted an opposite trend for the ‘searching’ measure. Searching showed moderate to strong correlations between internal situations (e.g., N vs. S for LF: Spearman’s; r = 0.769, p = 1.07 × 10^–6^) (Supplementary Table 1C). Thus, independent of its odor environment, larval searching was influenced by an individual’s satiety state. Two examples comparing N-EF with S-EF and N-LF with S-LF are shown (Fig. 2G, H). On the other hand, searching did not correlate between external situations (odor) for either satiety state considered. Thus the searching behavior of a starved larva in situation 1 did not correlate with the same starved larva’s searching behavior in situation 2 (Supplementary Table 1A, B).

Our data suggest that average measures of different behavior traits (activity and searching) and individual idiosyncrasies in that behavior are differently influenced by internal and external situations. Larval activity is stable across odor environments (external situations) given a particular satiety state (internal situation) but not stable across satiety states given an odor context. Larval searching has the opposite pattern: it is stable across satiety states given a particular odor environment but not stable across odors given a satiety state.

### The stability of olfactory behavior syndromes is not influenced by first-order olfactory sensory neuron activity

Peripheral olfactory neurons play critical roles in encoding internal and external information. Therefore, in experiment 3, we asked whether the idiosyncratic behavior could be influenced by first-order olfactory sensory neurons (OSNs). We compared the ‘activity’ of an individual larva between three situations: situation 1 (pre-exposure: lights OFF, 1 min.); situation 2 (during-exposure: lights ON, 1 min.); situation 3 (post-exposure: lights OFF, 1 min.) (Fig. 3A, B). For the control line and all seven OSNs tested, larval activity showed a high degree of correlation pre-, during, and post-OSN-activation (Fig. 3D–K; Supplementary Table 2). Thus, a larva with high activity pre-activation of an OSN tended to remain active during and after that OSN. While we tested only a subset of one-third of all larval OSNs, our results suggest that OSN activation does not influence the stability of a behavior syndrome.

### The stability of olfactory behavior syndromes is influenced by at least one group of antennal lobe local neurons

Several adaptive behaviors in insects originate in the activities of downstream local neurons and second-order projection in the antennal lobe^43^. Therefore, in experiment 4, we asked whether any downstream olfactory neurons could influence the stability of olfactory behavior syndromes. First, we temporally inactivated specific sets of neurons. Then, we compared the ‘activity’ and ‘shape’ of individual larvae before (situation-1) and after (situation-2) neuron inactivation. In all lines tested, including control, we observed no correlation in ‘activity’ (trait) between situation-1 and situation-2 (data not shown). The lack of correlation in ‘activity’ in contrast to Experiment 2 could be due to differences in the assay system. For example, a smaller circular arena rather than the larger square arena in Experiment 2 could affect the odor gradient’s shape and offer less surface area for the larva to crawl. These factors could affect the stability of ‘activity.’ We also cannot rule out the possibility that external temperature affects the stability of ‘activity.’ However, when we examined the “shape” trait, we found that one line (189Y) showed a positive correlation (R = 0.529, p = 0.0422) (Fig. 4E; Supplementary Table 3). Thus, a larva with a high value for ‘shape’ before 189Y neurons were inactivated maintained a high value for ‘shape’ after inactivation. Such a positive correlation was not observed in the control line (p = 0.0366, Fisher R-to-Z transform) or in any other lines tested (Fig. 4D–K; Supplementary Table 3). This data suggests that antennal lobe local neurons labeled by the 189Y-Gal4, when active, disrupt the stability of olfactory behavior syndromes.

## Discussion

We carried out four experiments that characterized the stability of olfactory behavior syndromes in the *Drosophila* larva. In experiment 1, we considered the stability of olfactory behavior syndromes during the short foraging stage of the third-instar *Drosophila* larva. We considered two different behavior syndromes in this experiment. Our results support the hypothesis that larval olfactory behavior syndromes are stable across a short developmental timescale (in the order of tens of minutes during the third-instar larval stage) (Fig. 1D, E). In experiment 2, we show that the stability of individual behavior traits is differently influenced by internal and external situations. For instance, larval ‘activity’ is stable across odor environments (external situations) given a particular satiety state (internal situation) but not stable across satiety states given an odor context. (Fig. 2C, E, F). Larval ‘searching’ has the opposite pattern: it is stable across satiety states given a particular odor environment but not stable across odors given a satiety state. (Fig. 2D, G, H). In experiments 3 and 4, we asked whether the stability of olfactory behavior syndromes can be influenced by peripheral olfactory neurons. In experiment 3, we tested seven different OSNs using an optogenetic assay. We found that OSN activity did not influence the stability of behavior syndromes (Fig. 3E–K). In experiment 4, we screened seven sets of neurons downstream of OSNs. We found that local neurons labeled by the 189Y-Gal4 disrupt the stability of olfactory behavior syndromes (Fig. 4E–K). Overall, these four experiments identified previously unknown factors that influence idiosyncratic behaviors in a crawling insect.

The third-instar *Drosophila* larval stage (a 24-h period that ranges from ~ 96 to ~ 120 h after egg laying) is characterized by dynamic modifications in behavior. The four experiments in this study focused on the behavior of early- to mid-third instar larvae that are primarily motivated by feeding^44^. Experiment 1 revealed idiosyncrasies in larval olfactory behavior even within this short developmental timeframe. This observation is significant because previous studies on the stability of behavioral syndromes have predominantly focused on evolutionary frameworks or developmental situations, e.g., behavior in the larval stage vs. adults^7,11–14^. Our results align with other noise-related studies, which show that noisy, transient neural mechanisms can generate idiosyncratic behaviors^15,16^. Since we focused on a single developmental stage of *Drosophila,* we could not compare the strength of the cross-situational stabilities between the third-instar larval stage and other developmental stages of the fruit fly.

Several studies have suggested that an animal’s external odor environments and internal states, such as hunger, influence their olfactory and food-search behaviors^38–42,45,46^. Experiment 2 focused on how external and internal situations influence idiosyncratic behaviors. First, we noted that third-instar larvae, given a particular satiety state (internal situation), exhibited higher activity in the presence of early- and late-ferment odors than the control diluent. On the other hand, starved larvae had significantly lower search values than non-starved larvae, given a particular odor environment (external situation). Since the ‘search’ parameter was based on track curvature and the number of head sweeps, a lower search value suggested that starved larvae had straighter tracks and fewer head sweeps. This result is consistent with what has been reported in many crawling and walking insects. *Drosophila* larvae, when starved, have smaller head sweeps and straighter trajectories^47^. When starved, many other crawling insect species have higher dispersal rates, often accompanied by significantly fewer head sweeps^47–51^. While a systematic search is most efficient when there is knowledge of food in the general vicinity, traveling further distances in a random direction might be a more efficient search strategy when food is sparse^52^. When we examined whether the larva’s internal and external states influenced the stabilities of these behavior traits, we found that a larva’s external situation influenced the stability of the ‘activity’ trait. In contrast, its internal situation influenced the stability of ‘searching.’ Previous studies have suggested that the type of odor in the environment affects larval run speed and run length toward or away from the odor (activity)^25,32^. Other studies have suggested that the larva’s satiety state affects whether a larva decides to engage in local searching or employ more efficient search strategies such as traveling further distances^47–52^. These findings complement previous studies about idiosyncrasies in an animal’s olfactory response and suggest a model in which internal and external states affect different features of idiosyncratic behavior, independent of statistical probabilities^19,22^. Whether the mechanisms influencing individual idiosyncrasies are dissociable or heritable remains to be studied. However, if behavior trait stabilities are heritable, they could explain why *D. melanogaster* strains derived from the same wild population display dramatic differences in their behavior responses to new odor environments^53–55^. They may also help us understand how and why starved animals show different degrees of search and exploratory behavior compared to non-starved animals^56–58^.

Experiments 3 and 4 explored olfactory circuit components that influence idiosyncratic behavior. We found that none of the seven first-order OSNs tested in this study influenced the stability of idiosyncrasies. We only tested a subset of all larval OSNs (7 out of 21). We note that the correlations in activity (pre-exposure vs. post-exposure) ranged from moderate (OSN::42b) to strong (e.g., OSN::47a). Therefore, based on our limited analysis of OSNs and the type of experimental design, we cannot rule out the possibility that some OSNs could influence the stability of idiosyncrasies. However, we found that a set of downstream neurons labeled by 189Y-Gal4 disrupt stability. In the *Drosophila* third-instar larval stage, 189Y-Gal4 drives expression in a subset of larval local neurons in the antennal lobe^59,60^. This role of influencing idiosyncratic behavior seems to be restricted to some antennal lobe local neurons and not all since 189Y-Gal4, and other Gal4 drivers tested in this study, such as NP3056-Gal4 and 449-Gal4 label non-overlapping subsets of local neurons^59^. At a basic level, this result suggested that at least some peripheral components of an olfactory system can modulate idiosyncratic behavior. This is not entirely surprising because fluctuations in the early stages of sensory systems propagate onward and influence the encoding of stimuli in other brain regions. For instance, manipulating the activity of individual glomeruli modulates a fly’s attraction to odors^61^. Thus, fly behavior responses are sensitive to small differences in peripheral activity.

Although these aspects improve our understanding of idiosyncratic behavior, they do not exhaust possible internal and external factors that influence idiosyncratic behavior nor the circuit mechanisms that control it. Indeed, past studies have proposed other factors that influence the stability of idiosyncratic behavior. These include hormonal^1,12,22^, age^7,12^, social^14,62^, and genetic factors^17,62,63^. While this study focused on peripheral neurons, central neurons in the brain can also play a role in influencing idiosyncratic behaviors. For instance, a group of *Drosophila* central complex cells (E-PG neurons) influence inter-individual variations in maintaining specific heading angles during sun-orientation experiments. When E-PG neurons were silenced, flies had smaller variances in their heading angles during menotaxis^64^. Similarly, another group of central complex cells (columnar PFNs) influence inter-individual variation in left or right bias during locomotion^65^. Establishing appropriate behavior paradigms in the larvae, such as in this study or in the adult fly^22^, will allow us to investigate the influence of other internal and external factors and specific molecular and neural mechanisms within olfactory circuits that could modulate the degree of idiosyncratic behavior. In future studies, we must consider the various sensory conditions that preserve or disrupt behavior syndromes. For instance, do specific attractive and aversive cues antagonistically affect behavior syndromes? Are these observations generalizable to other strains of *D. melanogaster* and other *Drosophila* species? Ultimately, understanding idiosyncratic behavior structures and their mechanisms is critical to understanding how circuit function influences animal behavior.

Why should a population of animals maintain a range of idiosyncratic behaviors? The range of behavior syndromes in an animal and the degree of their stability have implications for species’ survival. If behavior idiosyncrasies are heritable, they provide a substrate for evolution by natural selection. It could explain how some *Drosophila* species have evolved specialized behavior responses to certain odorants^53,66,67^. If not heritable, they may at least alter evolutionary dynamics by providing a behavior buffer for populations experiencing drastic ecological challenges^68^. By displaying a range of phenotypes, even within a fixed genotype, animals inadvertently employ a bet-hedging strategy. Such a strategy guarantees that individuals are always well-adapted to any situation, even in the face of unpredictable environmental fluctuations. Thus, a goal of maintaining a range of idiosyncratic behavior responses among individuals of a population could be to maintain or increase the species’ overall fitness. The degree of behavior syndrome stability matters, too. For instance, a very stable behavior syndrome restricts behavior plasticity, leading to suboptimal behaviors and effects on species’ long-term survival^1,2,7,69^.

Our study highlights the importance of studying idiosyncrasies in animal behavior. Past fly studies have attempted to connect peripheral activity patterns in the olfactory circuit to behavioral responses to odors^27,33,61,70–72^. Despite several elegant approaches, we currently lack a unified odor coding model which relates circuit activity to behavior response. This could be due to a traditional focus on averaging behavior measures in populations of flies. While such an approach has yielded important insights over decades of research, an integral component of the neuro-behavior relationship is typically discarded. Determining how behavior structures are influenced by small variations in the function of neural circuits is crucial to understanding how behaviors are generated by the nervous system.

## Supplementary Information

Supplementary Information.

## Data Availability

Key resources used in this study are listed in the “Materials and methods” section. Further information and requests for resources and reagents, raw data, and software codes generated within the present study should be directed to and will be fulfilled by the Lead Contact, Dennis Mathew (dennsimathew@unr.edu).
