# Supplementary material for: Stability of olfactory behavior syndromes in the Drosophila larva
Source: Sci Rep. 2023 Feb 10;13:2398. doi: 10.1038/s41598-023-29523-x (PMC9918538; doi:10.1038/s41598-023-29523-x)
Supplement: Supplementary file 1 — Supplementary Information. [file 41598_2023_29523_MOESM1_ESM.pdf]

SUPPLEMENTARY MATERIAL

Stability of Olfactory Behavior Syndromes in the *Drosophila* Larva

Seth R. Odell[1], Nicholas Zito[1], David Clark[1], and Dennis Mathew[1,2 *]

[1]Integrative Neuroscience Program, University of Nevada, Reno, NV 89557; USA
[2]Department of Biology, University of Nevada, Reno, NV 89557; USA

***Correspondence**:
Corresponding author: Dr. Dennis Mathew
Email: dennismathew@unr.edu
Address: 1664 N. Virginia St., MS: 0314, University of Nevada, Reno, NV 89557; USA

Supplementary Figure 1: Principal component analyses of activity and dispersal behaviors. (**A**) Principal component analysis of three measures: % time stopped, mean run speed, and % plate explored. Black dots indicate larvae in situation 1. Pink dots indicate larvae in situation 2. The table shows loads of each variable on the PC, latency, and total variance explained by each PC. Variable loads are represented on the graph by black lines radiating from the origin and labeled. High values of PC-1 (Activity) indicate a larva with a higher run speed, explored more of the plate and spent less time at a stop. (**B**) Principal component analysis of two measures: max. distance from the center and time to leave the center. The table shows loads of each variable on the PC, latency, and total variance explained by each PC. Variable loads are represented on the graph by black lines radiating from the origin and labeled. High values of PC-1 (Dispersal) indicate a larva that more rapidly left the center of the plate and traveled further from the center of the plate.

Supplementary Figure 2: Principal component analysis of activity and search behaviors in six situations. Principal component analysis of five measures: mean run speed, mean run length, total curvature, head sweeps, and % time stopped. PC-1 axis represents 'activity.' PC-2 axis represents 'search' behavior. Black dots indicate situation 1 (no odor). Red dots indicate situation 2 (early ferment odor). Blue dots indicated situation 3 (late ferment odor). For the sake of readability, the same analysis has been represented twice to separately visualize data for non-starved (solid dots) (**A**) and starved larvae (empty dots) (**B**). (**C**) The table shows loads of each variable on the PC, latency, and the total variance explained by each PC. Variable loads are represented on the graph by black lines radiating from the origin and labeled. High values of PC-1 (Activity) indicate a larva with a higher run speed and run length and spent less time at a stop. High values of PC-2 (Search) indicate a larva that had a higher total number of head sweeps and had a more meandering track) high values for curvature – overall length/displacement).

Supplementary Figure 3: Principal component analysis of activity behavior during OSN activation. Principal component analysis of three measures: % time stopped, mean run speed, and % plate explored. PC-1 axis represents 'activity.' Black dots indicate control larvae. Color dots represent transgenic larvae in which a different OSN expresses *ChRhodopsin*: OSN::Or42b (red); OSN::Or45b (medium blue); OSN::Or7a (brown); OSN::Or42a (light blue); OSN::Or45a (green); OSN::Or47a (dark blue); OSN::Or67b (purple). For the sake of readability, the same analysis has been represented thrice to separately visualize data for larvae in situation 1 (pre-OSN activation) (open dots) (**A**), data for larvae in situation 2 (during OSN activation) (solid filled dots) (**B**), and data for larvae in situation 3 (post-OSN activation) (translucent filled dots) (**C**). (**D**) The table shows loads of each variable on the PC, latency, and the total variance explained by each PCA. Variable loads are represented on the graph by black lines radiating from the origin and labeled. High values of PC-1 (Activity) indicate a larva with a higher run speed, explored more of the plate and spent less time at a stop.

Supplementary Figure 4: Principal component analysis of behavior during downstream neuron inactivation. Principal component analysis of five measures: mean run length, mean run speed, the total number of sweeps, total curvature, and % time stopped. PC-1 axis represents 'activity.' PC-2 axis represents 'search.' Black dots represent the parent control line. Color dots represent transgenic larvae in which a different set of downstream neurons expresses a temperature-sensitive *shibire* construct: 189Y (Yellow), NP3056 (Light Blue), 421 (Green), 449 (Gray), Keystone (Dark Blue), SEZ (Orange), ACJ6 (pink). (**A**) Data for larvae at the permissive temperature, 25 C (filled dots). (**B**) Data for larvae at the restrictive temperature, 35 C (open dots). (**C**) The table shows the variable load of the PC, latency, and the total variance explained by each PC. Variable loads are represented by blank lines radiating from the origin and labeled. High values of PC-1 (Activity) indicate a larva spent little time at a stop. High PC-2 (Search) values indicates a higher number of sweeps and higher curvature, with a lower run speed and run lengths.

Supplementary Table 1: Correlation coefficients: Coefficients for Activity measures are Pearson's correlation. Coefficients of Searching measures are Spearman's correlation. The average value was calculated by transforming each correlation coefficient using the Fisher R-to-Z transform, averaging the values, and then back transforming. (* p<0.025; **p<0.001; ***p<0.0001). (**Supplementary Table 1A**) Correlation coefficients for non-starved state comparisons**.** The table shows the correlation coefficients and significance values between non-starved larvae in the presence of each odor (N-EF, N-LF, N-PO). (**Supplementary Table 1B**) Correlation coefficients for starved state comparisons. The table shows the correlation coefficients and significance values between the starved larvae in the presence of each odor (S-EF, S-LF, S-PO). (**Supplementary Table 1C**) Correlation coefficients for odor comparisons. The table shows the correlation coefficients and significance values between the non-starved and starved larvae in the presence of each odor.

Supplementary Table 2: Correlation coefficients for pre-, during, and post-activation of larval OSNs. The table shows the correlation coefficients and significance values between Activity scores in three situations: pre-, during, and post-activation of OSNs. This is shown for control larvae and seven other genotypes in which a different OSN expresses *ChRhodopsin*. All correlation coefficients are Pearson's correlation.

Supplementary Table 3: Correlation coefficients for larval olfactory neuron screen. The table shows the correlation coefficients and significance values between permissive and restrictive temperatures for both activity and shape behaviors. All correlations are Spearman's correlations.

Supplementary figure 1

A

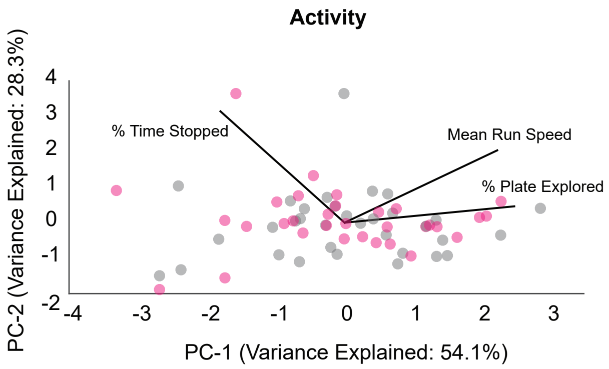

	PC-1	PC-2	PC-3
% Time Stopped	-0.478	0.832	0.281
Mean Run Speed	0.588	0.541	-0.602
% Plate Explored	0.653	0.122	0.747
Latency	1.62	0.849	0.529
Variance Explained	54.1%	28.3%	17.6%

B

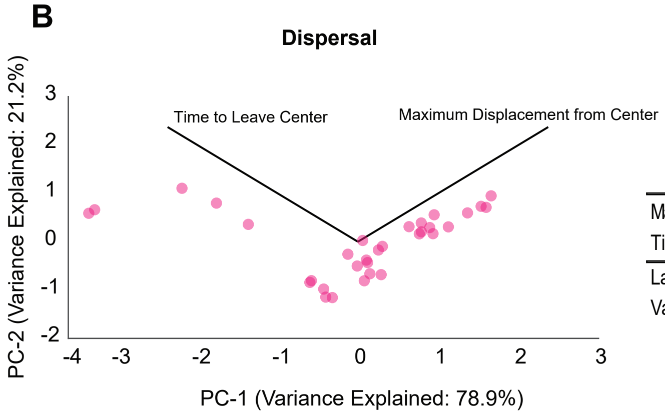

	PC-1	PC-2
Maximum Distance from Center	0.707	0.707
Time To Leave Patch	-0.707	0.707
Latency	1.58	0.423
Variance Explained	78.9%	21.2%

Supplementary figure 2

A

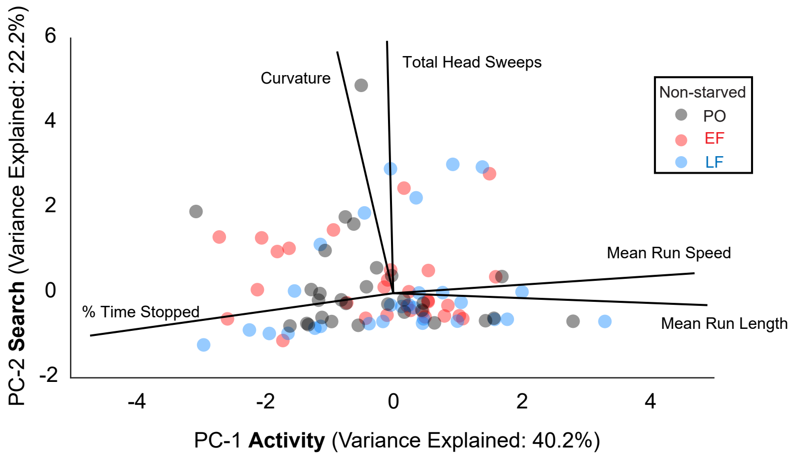

B

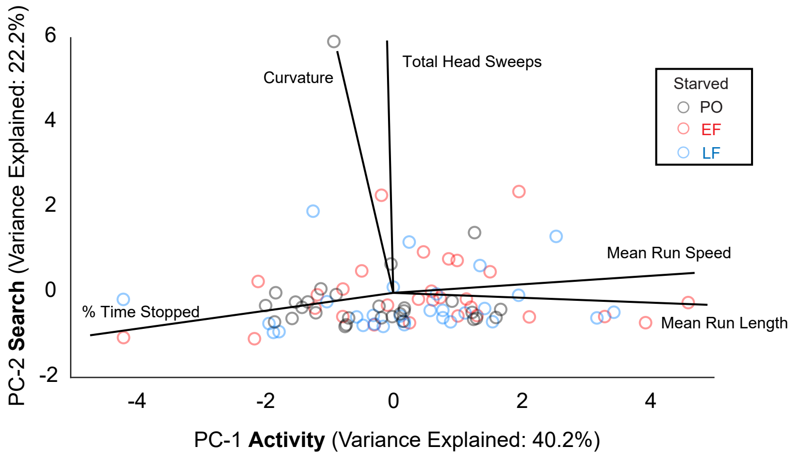

C

	**PC-1**	**PC-2**	**PC-3**	**PC-4**	**PC-5**
Mean Run Speed	0.565	0.0566	0.173	0.703	-0.392
Mean Run Length	0.589	-0.0338	-0.0311	-0.0130	0.807
Curvature	-0.105	0.685	0.705	-0.0705	0.132
Total Sweeps	-0.0117	0.715	-0.687	0.128	0.0141
% Time Stopped	-0.568	-0.120	0.0230	0.696	0.422
Latency	2.01	1.11	0.896	0.520	0.460
Variance Explained	40.2%	22.2%	17.9%	10.4%	9.22%

Supplementary figure 3

A

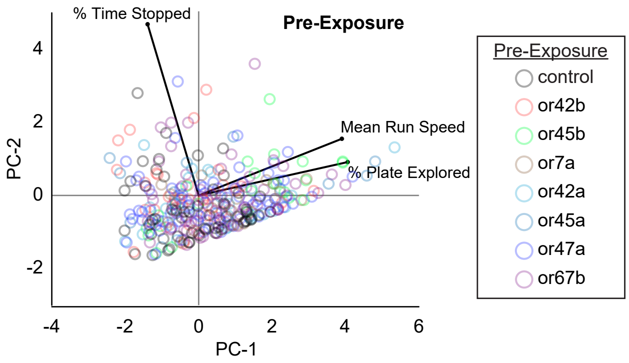

B

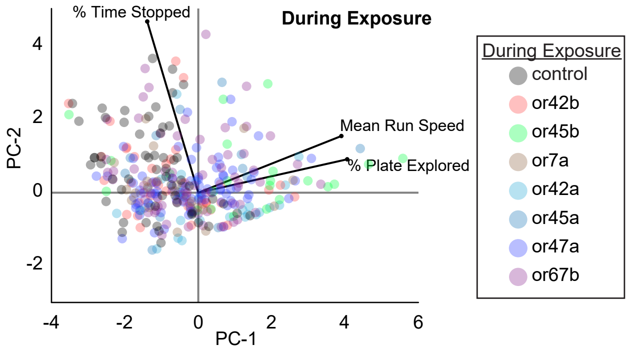

C

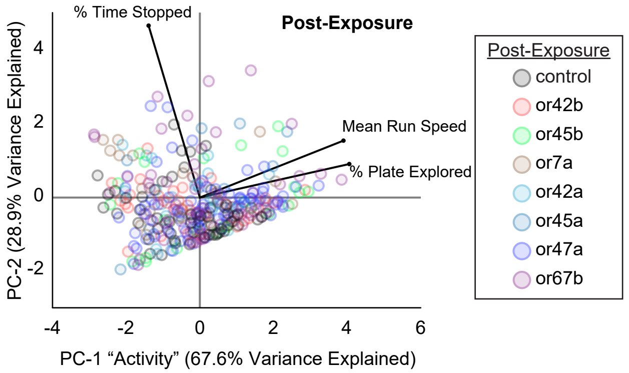

D

	**PC-1**	**PC-2**	**PC-3**
**Mean Run Speed**	0.653	0.308	-0.691
**% Time Stopped**	-0.347	0.934	0.0885
**% Plate Explored**	0.672	0.182	0.718
**Latency**	2.03	0.866	0.107
**% Variance Explained**	67.6	28.9	3.6

Supplementary figure 4

A

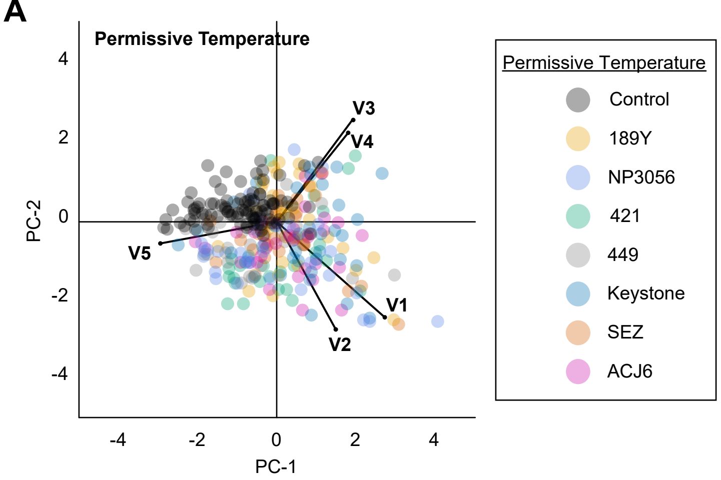

B

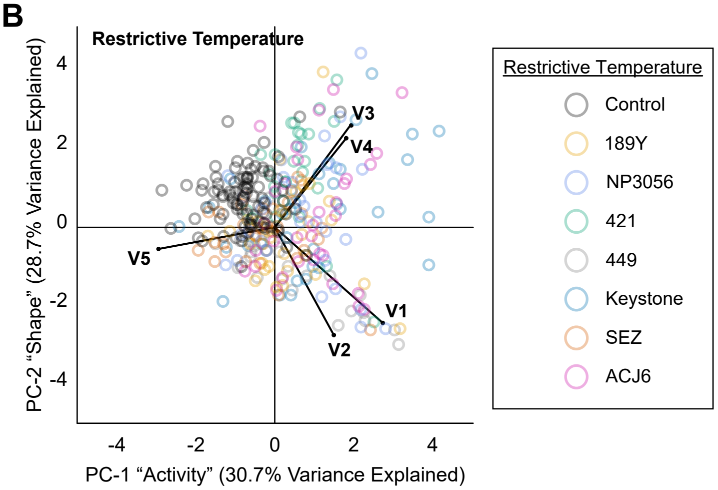

C

	PC-1	PC-2	PC-3	PC-4	PC-5
**Mean Run Length (V1)**	0.542	-0.480	-0.126	-0.150	0.661
**Mean Run Speed (V2)**	0.295	-0.542	0.582	0.244	-0.470
**Total Sweeps (V3)**	0.383	0.513	0.196	0.698	0.254
**Curvature (V4)**	0.357	0.448	0.492	-0.655	-0.0221
**% Time Stopped (V5)**	-0.587	-0.109	0.604	0.0409	0.526
**Latency**	1.54	1.43	0.912	0.688	0.431
**% Variance Explained**	30.7	28.7	18.2	13.8	8.61

Supplementary Table 1

Supplementary Table 1A: Correlation coefficients for non-starved state comparisons.

	Average		N-EF vs N-LF	N-EF vs N-PO	N-LF vs N-PO
Activity	0.535	r	0.535*	0.468	0.594**
		p	0.00725	0.0236	0.00413
Searching	0.0286	r	-0.405	0.223	0.281
		p	0.0527	0.295	0.179

Supplementary Table 1B: Correlation coefficients for starved state comparisons.

	Average		S-EF vs S-LF	S-EF vs S-PO	S-LF vs S-PO
Activity	0.600	r	0.763***	0.560*	0.416*
		p	1.32e-5	0.00720	0.0498
Searching	0.124	r	-0.0340	0.0665	0.328
		p	0.775	0.861	0.115

Supplementary Table 1C: Correlation coefficients for odor comparisons.

	Average		N-EF vs S-EF	N-LF vs S-LF	N-PO vs S-PO
Activity	0.305	r	0.351	0.365	0.194
		p	0.0924	0.0846	0.354
Searching	0.629	r	0.534*	0.769***	0.542*
		p	0.00725	1.32e-5	0.00725

Pearson's correlation for 'activity' measures

Spearman's correlation for 'searching' measures

Values in red indicate significant correlation: * $p < 0.05$; ** $p < 0.01$; *** $p < 0.001$

Supplementary Table 2

Supplementary Table 2: Correlation coefficients for pre-, during, and post-activation of larval OSNs.

OSN	Exposure	Exposure	r	p
control	Pre	During	0.584***	2.79e-6
control	Pre	Post	0.391**	0.00310
control	During	Post	0.620***	5.28e-7
OSN::Or7a	Pre	During	0.572**	0.0084
OSN::Or7a	Pre	Post	0.619**	0.00360
OSN::Or7a	During	Post	0.875***	4.34e-7
OSN::Or42a	Pre	During	0.690***	3.52e-5
OSN::Or42a	Pre	Post	0.512**	0.00450
OSN::Or42a	During	Post	0.703***	2.09e-5
OSN::Or42b	Pre	During	0.568**	1.65e-4
OSN::Or42b	Pre	Post	0.340*	0.0342
OSN::Or42b	During	Post	0.717***	2.87e-7
OSN::Or45a	Pre	During	0.783***	9.21e-4
OSN::Or45a	Pre	Post	0.886***	2.43e-5
OSN::Or45a	During	Post	0.755**	0.00180
OSN::Or45b	Pre	During	0.733***	4.62e-5
OSN::Or45b	Pre	Post	0.662***	4.29e-4
OSN::Or45b	During	Post	0.546**	0.00580
OSN::Or47a	Pre	During	0.828***	2.63e-21
OSN::Or47a	Pre	Post	0.671***	9.98e-12
OSN::Or47a	During	Post	0.704***	3.13e-13
OSN::Or67b	Pre	During	0.622***	1.19e-8
OSN::Or67b	Pre	Post	0.630***	6.50e-9
OSN::Or67b	During	Post	0.728***	1.37e-12

Pearson's correlation.

Values in red indicate significant correlation: * $p < 0.05$; ** $p < 0.01$; *** $p < 0.001$

Supplementary Table 3

Supplementary Table 3: Correlation coefficients for larval olfactory neuron screen.

Line	Behavior	r	p
control	Activity	0.241	0.0630
control	Shape	0.130	0.426
189Y	Activity	0.114	0.766
189Y	Shape	0.529*	0.0422
NP3056	Activity	0.168	0.575
NP3056	Shape	-0.0154	0.933
421	Activity	-0.110	0.766
421	Shape	0.288	0.364
449	Activity	0.0936	0.766
449	Shape	0.435	0.0633
Keystone	Activity	0.435	0.0538
Keystone	Shape	0.45	0.0538
SEZ	Activity	0.180	0.575
SEZ	Shape	-0.670	0.805
ACJ6	Activity	-0.232	0.426
ACJ6	Shape	0.13	0.426

Spearman’s correlation for all measures

Values in red indicate significant correlation: * $p < 0.05$

Supplementary Table 5: Key Resource Table

REAGENT or RESOURCE	SOURCE	IDENTIFIER
**Chemicals, Peptides, and Recombinant Proteins**		
Ethyl Acetate	Sigma-Aldrich	319902 CAS: 141-78-6
Acetal	Sigma-Aldrich	200220 CAS: 105-57-7
Acetic Acid	Sigma-Aldrich	320099 CAS: 64-19-7
Paraffin Oil	Sigma-Aldrich	76235 CAS: 8012-95-1
Agarose	Genesee Scientific	20-102GP
All-trans Retinal	Sigma-Aldrich	R2500 CAS: 166-31-4
**Deposited Data**		
Raw and Analyzed Data	This Paper	Available upon request
**Experimental Models: Organisms/Strains**		
D. melanogaster: Canton-S	John Carlson	
D. melanogaster: uas-IVS-CsChrimson<br>w [1118]; P{y [+t7.7] w [+mC]=20XUAS-IVS-CsChrimson.mVenus}attP40	Bloomington Fly Stocks	RRID:BDSC_55135
D. melanogaster: or7a-Gal4<br>w [*]; Sp/CyO; Or7a-GAL4	John Carlson	
D. melanogaster: or42a-Gal4<br>w [*]; P{w [+mC]=Or42a-GAL4.F}48.3B	Bloomington Fly Stocks	RRID:BDSC_9970
D. melanogaster: Or42b-Gal4<br>w [*]; P{w [+mC]=Or42b-GAL4.F}64.3	Bloomington Fly Stocks	RRID:BDSC_9971
D. melanogaster: Or45a-Gal4<br>w [*]; P{w [+mC]=Or45a-GAL4.F}43.5	Bloomington Fly Stocks	RRID:BDSC_9976
D. melanogaster: Or47a-Gal4<br>w [*];Or47a-Gal4; Dr/TM3	John Carlson	
D. melanogaster: Or67b-Gal4<br>w [*];Sp/CyO; Or67b-GAL4/TM3	John Carlson	
**Software and Algorithms**		
MATLAB v 2019b	Mathworks	RRID:SCR_001622
Statistica v13.5	Statsoft	RRID: SCR_014213
R v3.3.1	www.r-project.org	RRID:SCR_001905
Tracking Code	This Paper	Available upon request
